# The role of cytoreductive nephrectomy in metastatic renal cell carcinoma in immune-oncology era (SEVURO-CN): study protocol for a multi-center, prospective, randomized trial

**DOI:** 10.1186/s13063-024-08234-2

**Published:** 2024-07-03

**Authors:** Jee Soo Park, Jongchan Kim, Jinhyung Jeon, Jongsoo Lee, Won Sik Jang, Seung Hwan Lee, Woong Kyu Han, Young Deuk Choi, Kyo Chul Koo, Kang Su Cho, Byung Ha Chung, Won Sik Ham

**Affiliations:** 1 Department of Urology and Urological Science Institute, Yonsei University College of Medicine, Seoul, South Korea; 2 Department of Urology, Yongin Severance Hospital, Yonsei University Health System, Seoul, South Korea; 3 Department of Urology, Gangnam Severance Hospital, Yonsei University College of Medicine, Seoul, South Korea

**Keywords:** Renal cell carcinoma, Cytoreductive nephrectomy, Ipilimumab, Nivolumab, Immune checkpoint inhibitors, Kidney neoplasms, Prospective studies

## Abstract

**Background:**

The role of cytoreductive nephrectomy (CN) in the treatment of metastatic renal cell carcinoma (mRCC) remains unclear in the immuno-oncology (IO) era. The results of two randomized trials, CARMENA and SURTIME, questioned the role and timing of CN. However, despite the latest advances in the systemic treatment of mRCC, previous trials have only used targeted therapy, and no studies have fully investigated the role of CN in immune checkpoint inhibitor (CPI) settings, and there is an urgent need for future studies to better define the role and timing of CN.

**Methods:**

This study is an open-label, multi-center, parallel, prospective, randomized, interventional clinical study to evaluate the efficacy of CN in combination with CPIs in mRCC patients with International mRCC Database Consortium (IMDC) intermediate- and poor-risk. Synchronous mRCC patients with ≤ 3 IMDC risk features will be randomly allocated to three groups (1, upfront CN; 2, deferred CN; and 3, systemic therapy [ST] only). For ST, the nivolumab plus ipilimumab combination regimen, one of the standard regimens for intermediate- and poor-risk mRCC, is chosen. The primary endpoint is overall survival. The secondary endpoints are progression-free survival, objective response rate, number of participants with treatment-related adverse events, and number of participants with surgical morbidity. We will analyze the genetic mutation profiles of the tumor tissue, circulating tumor DNA, urine tumor DNA, and tumor-infiltrating lymphocytes. The gut and urine microbial communities will be analyzed. The study will begin in 2022 and will enroll 55 patients.

**Discussion:**

This study is one of the few prospective randomized trials to evaluate the benefit of CN in the treatment of synchronous mRCC in the IO era. The SEVURO-CN trial will help identify the role and timing of CN, thereby rediscovering the value of CN.

**Trial registration:**

ClinicalTrials.gov, NCT05753839. Registered on 3 March 2023.

**Supplementary Information:**

The online version contains supplementary material available at 10.1186/s13063-024-08234-2.

## Background

Annually, over 75,000 individuals are newly diagnosed with renal cell carcinoma (RCC) in the USA, and its incidence is increasing [1]. Although the majority of RCC are diagnosed as localized cases, approximately one-third of patients have synchronous metastasis [2]. Nearly 14,000 cancer-specific deaths have been reported in the USA [1], with 5-year survival rates for metastatic renal cell carcinoma (mRCC) being quite low (0–20%), establishing it as a deadly disease [3–6].

Based on two randomized trials, cytoreductive nephrectomy (CN) was previously considered the standard treatment for the management of patients with mRCC with a good performance status [7, 8]. However, these studies were performed in the era of interferon therapy [7, 8], and dramatic advancements in systemic therapies have questioned the utility of CN. Two prospective randomized trials, CARMENA [9] and SURTIME [10], changed the standard first-line treatment for mRCC by downsizing the role of CN.

However, recently updated analyses of the CARMENA trial have suggested that some patients still benefit from CN [11]. Furthermore, the development of immune checkpoint inhibitors (ICIs) has revolutionized the treatment landscape [12], disrupting the treatment paradigm for mRCC and further complicating the definition of the role of CN. ICIs are now the standard of care for patients with intermediate- and poor-risk diseases. However, the CARMENA and SURTIME trials were performed in the tyrosine kinase inhibitor (TKI) era [13]; therefore, current clinical data on ICIs are limited. The role of CN in patients with high-risk localized tumors and favorable-to-intermediate-risk metastatic tumors falls into a gray zone [14]. Therefore, rather than a black-and-white approach, a combination of surgery and systemic therapy is likely to be beneficial for these patients [15].

The theoretical benefits of CN remain unclear, but the postulated mechanisms include the removal of the immunological sink, reduction in potential interaction between the primary tumor and its metastatic sites, and removal of possible de novo metastatic disease that stems from the renal primary [14, 16].

Two ongoing phase 3 randomized clinical trials (RCTs), PROBE (NCT04510597) and NORDICSUN (NCT03977571), are currently investigating the role of deferred versus no CN, and neither trial has included an upfront CN arm. Furthermore, no studies have been presented regarding the association between the analysis of clinical samples from the tumor tissue, blood, urine, and feces, and clinical parameters to develop biomarkers. Finally, a patient selection model for upfront CN in immuno-oncology (IO) era has never been presented.

### Objectives

The primary aim of this study is to investigate the role of CN in overall survival (OS). Our secondary aim is to investigate the role of CN in the progression-free survival (PFS), objective response rate (ORR), number of participants with treatment-related adverse events, and number of participants with surgical morbidity.

Furthermore, we will collect samples (tumor tissue, blood, urine, and feces) not only to explore the effects of CN in the tumor immune microenvironment (TIME) by analyzing genetic mutations and microbiome profiles but also to establish a biobank for future studies that search for biomarkers focusing on identifying survival outcomes, therapeutic responses, and patient selection.

## Methods and analysis

### Study design

The current study is a prospective, randomized, open-label, multicenter, interventional, superiority clinical trial with a parallel-arm design, and will be conducted at the Yonsei University Health System (YUHS), which includes Severance Hospital, Gangnam Severance Hospital, and Yonsei Severance Hospital. After the startup phase, we will expand the study to other South Korean centers. The study was registered at ClinicalTrials.gov (NCT05753839). This trial complied with Standard Protocol Items: Recommendations for Interventional Trials (SPIRIT) guidelines. The SPIRIT checklist can be found in Additional file 1. All data will be anonymized and collected on structured case reporting forms.

This study will investigate whether upfront or deferred CN improves oncological outcomes (OS and PFS) in patients with synchronous mRCC and ≤ 3 International mRCC Database Consortium (IMDC) risk features compared to immune checkpoint inhibitors (nivolumab plus ipilimumab combination) alone.

To understand the role of CN in immuno-oncology, we will collect and analyze clinical data and samples (tumor tissue, blood, urine, and stool samples).

### Primary endpoint

The primary endpoint is OS (in months) compared between patients receiving upfront CN, deferred CN, and ICIs only.

### Recruitment and consent

Overall patient recruitment will be supervised at Severance Hospital, where the project leader and the research center are located, and each center will recruit patients under the guidance of Severance Hospital. A multidisciplinary team composed of urologists, medical oncologists, and radiologists will determine patient selection under strict inclusion and exclusion criteria and regularly update these criteria as necessary.

Patients diagnosed with synchronous mRCC with clear-cell components and no prior therapy will be recruited. The study information will be provided to the patients during the first consultation and asked for their participation the next day, giving them sufficient time to consider. During patient participation, the most current written informed consent approved by the Ethics Committee (Additional file 2) will be obtained before any project-specific assessment or procedure. No study-related activities will begin before the informed consent form is signed. The participants will be allowed to retire at any point during the study. This information is clearly stated in the consent form.

### Compensation

Although no compensation will be provided for patients in this study, future patients would benefit greatly. Participation will be voluntary, and the management of patients will comply with current standards of care.

### Patients

Patient selection is based on the following inclusion, dropout, and exclusion criteria.

#### Inclusion criteria

- Core needle biopsy-proven metastatic renal cell carcinoma—only clear cell histologic subtypes are acceptable.
- Synchronous metastatic renal cell carcinoma with a primary tumor in the kidney.
- The patient must be willing to provide their human-derived materials.
- Age ≥ 19 years.
- Signed written informed consent must be obtained from all patients prior to any study-specific procedures.
- The patient must be willing and able to comply with the protocol.
- Measurable disease as per response evaluation criteria for solid tumors (RECIST) v 1.1.
- Life expectancy of greater than 4 months.
- Patients with more than one prognostic factor by the IMDC criteria (intermediate- or poor-risk group).
- Patients indicated nivolumab/ipilimumab, according to the recommendations by the national health authorities. The prescription of nivolumab/ipilimumab under the circumstances of the study is considered standard treatment.
- Karnofsky Performance status ≥ 70.
- Females with a negative serum pregnancy test unless childbearing potential can be otherwise excluded (postmenopausal, hysterectomy, or oophorectomy) and not lactating.
- Fertile women of childbearing potential (< 2 years after last menstruation) and men must use effective means of contraception (oral contraceptives, intrauterine contraceptive device, barrier method of contraception in conjunction with spermicidal jelly or surgical sterilization).
- The required laboratory values are as follows:
  - Adequate bone marrow function (Absolute neutrophil count > 1500/mm3, platelets > 100 × 103/µl, hemoglobin > 10.0 g/dL)
  - International normalized ratio (INR) ≤ 1.2 × upper limit of normal (ULN)
  - Adequate hepatic function (bilirubin ≤ 1.5 × ULN, ALAT ≤ 2.5 × ULN)
  - Adequate kidney function (eGFR > 35 mL/min)

#### Dropout criteria

If a subject fulfills any of the following dropout criteria, they will be discontinued from the middle of the study:

- Patients who do not want to participate in the study.
- Patients who fall into the exclusion criteria during the study (such as the patients who need for starting systemic corticosteroids, antibiotics, investigational drugs, etc., during the study).
- Patients with severe side effects during the treatment and treatment can no longer be continued according to the schedule.
- Patients who do not adhere to the protocol of the study.

#### Exclusion criteria

If a subject fulfills any of the following exclusion criteria, they may not be included:

- Prior systemic treatment for mRCC.
- Major surgical procedure, open surgical biopsy, or significant traumatic injury within 28 days before enrollment.
- Other cancer within 5 years.
- Clinically significant (i.e., active) cardiovascular disease, for example, cerebrovascular accidents (< 6 months before inclusion), myocardial infarction (< 6 months before inclusion), unstable angina, and New York Heart Association (NYHA) grade II or greater congestive heart failure.
- No symptomatic brain metastasis requiring systemic corticosteroids (> 10 mg daily prednisone equivalent).
- Recent (within 30 days prior to inclusion) treatment with another investigational drug or participation in another investigational study.
- Any active or recent history of a known or suspected autoimmune disease or recent history of a condition requiring systemic corticosteroids (> 10 mg daily prednisone equivalent) or other immunosuppressive medications, excluding inhaled and topical steroids. Patients with vitiligo, type I diabetes mellitus, or residual hypothyroidism due to autoimmune thyroiditis requiring hormone replacement alone, and psoriasis not requiring systemic treatment are permitted to enroll.
- Oral or i.v. antibiotics administered 14 days prior to initiation of systemic therapy.
- Any positive test for hepatitis B- or C-virus indicating acute or chronic infection.
- Known hypersensitivity to monoclonal antibodies.
- Known history of testing positive for human immunodeficiency virus (HIV) or known acquired immunodeficiency syndrome (AIDS).
- Patients disagreeing to provide their human-derived materials.
- Vulnerable patients (such as children, prisoners, pregnant women, mentally disabled persons, or economically or educationally disadvantaged persons).
- Patients who cannot read and understand the consent form (such as individuals who are illiterate or foreigners).

#### Group-specific criteria

Group A: Upfront CN: cytoreductive nephrectomy ± metastasectomy, followed by induction therapy with nivolumab plus ipilimumab, and maintenance therapy with nivolumab.

Group B: Deferred CN: cytoreductive nephrectomy ± metastasectomy after induction therapy with nivolumab plus ipilimumab, followed by maintenance therapy with nivolumab.

Group C: No surgery: induction therapy with nivolumab plus ipilimumab followed by maintenance therapy with nivolumab.

### Study timeline

After completing the recruitment procedure, clinical and pathological data were collected (Table 1).

**Table 1:** Clinical and pathological data

Clinical data				Pathological data
Baseline	Surgery-related	ICI therapy-related	Oncological outcome	
Age	OR note (including any intraoperative events)	Dosage	Overall survival	Pathological TNM staging
Sex	EBL	Cycle	Progression-free survival	Cell type
Height	Surgical morbidity (according to CDC)	Toxicities (graded by CTCAE v5.0)	Objective response rate (according to RECIST v1.1)	ISUP nuclear grade
Weight		Total administration periods		
BMI		Cause of discontinuation		
BSA				
KPS				
IMDC risk feature				
Clinical TNM staging				
Metastatic sites				

*BMI* body mass index, *BSA* body surface area, *CDC* Clavien–Dindo classification, *CTCAE* Common Terminology Criteria for Adverse Events, *EBL* estimated blood loss, *IMDC* International mRCC Database Consortium, *ISUP* International Society of Urologic Pathologists, *KPS* Karnofsky Performance Status, *OR* operating room, *RECIST* Response Evaluation Criteria for Solid Tumors

**Fig. 1:**
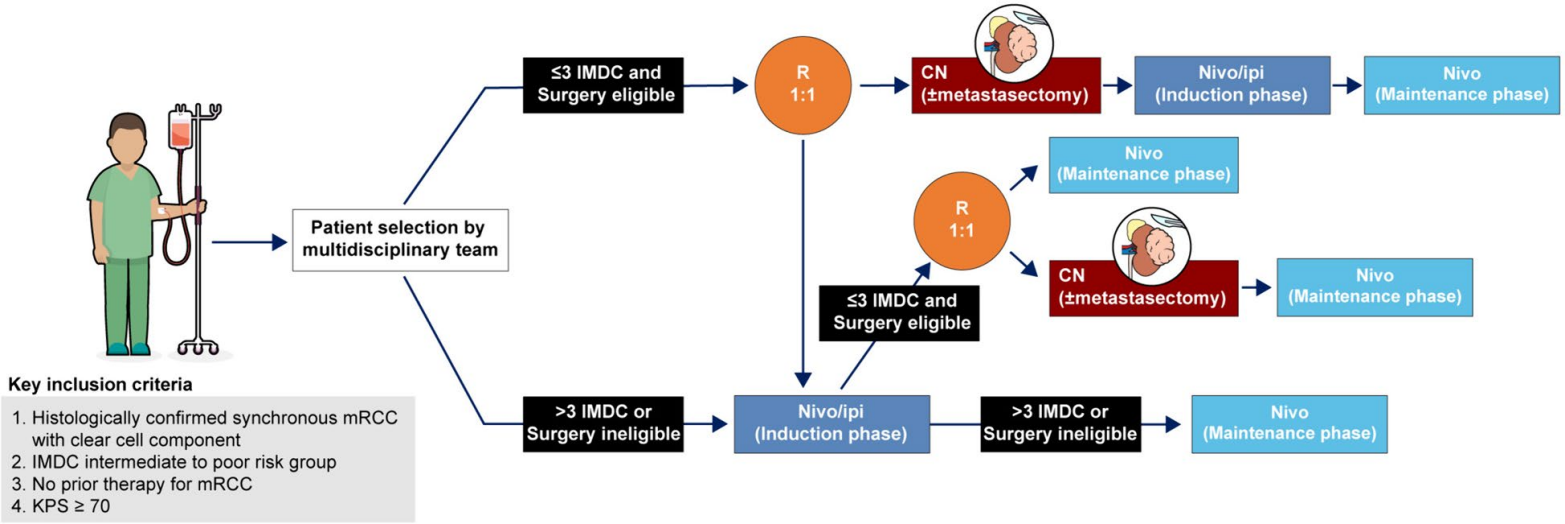
Study flowchart. CN, cytoreductive nephrectomy; IMDC, International mRCC Database Consortium; Ipi, ipilimumab; mRCC, metastatic renal cell carcinoma; Nivo, nivolumab; KPS, Karnofsky performance status

A flowchart of the study is shown in Fig. 1. Patients will be evaluated for eligibility for surgery. If they are not eligible for surgery, they will start ICI therapy (induction phase). Then, they will be assessed for whether they are eligible for surgery. If not, they will continue with ICI therapy (maintenance phase) (Group C). If they are eligible for surgery, they will be randomly allocated to the deferred CN group (Group B) or ICIs only. If they are eligible for surgery at the first visit, they will be randomly allocated to the upfront CN Group A (group A) or the deferred CN group (Group B). Patients will be randomly allocated in a 1:1 ratio using a computer-generated allocation sequence by a local project nurse. The randomization will be performed on an institutional basis to prevent bias.

Candidates for CN are patients with good performance status (Karnofsky performance status ≥ 80%) and less than three IMDC risk features and whose primary tumor could be surgically removed. Metastasectomy can be performed when the metastatic sites can be surgically removed. All patients eligible for ICI therapy will receive four cycles of intravenous nivolumab (3 mg/kg) plus ipilimumab (1 mg/kg) every 3 weeks as the induction phase, followed by a response evaluation. After the induction phase, intravenous nivolumab (3 mg/kg) will be administered 6 times every 2 weeks as the maintenance phase, followed by response evaluation. Nivolumab monotherapy will be maintained for a maximum of 2 years for those without adverse side effects. During ICI therapy, adverse events will be evaluated once per cycle. Patients will discontinue ICI therapy when they develop serious adverse events, including grade 3 or worse, according to the Common Terminology Criteria for Adverse Events version 5.0 (CTCAE v 5.0). In the upfront CN group, the induction phase will be within 6 weeks of surgery, whereas in the deferred CN group, surgery will be performed within 6 weeks of the induction phase. The study will be conducted over a period of 5 years.

### Sample collection

Tumor tissue, blood, urine, and stool specimens for translational biomarker research will be sampled at the baseline visit, during surgery, 3 months after induction therapy, and 3 months after maintenance therapy. Embedded future studies will have their specific protocol and will obtain specific approval from the ethics committee separately. The tumor tissue from the participants will be obtained from a core needle biopsy sample (20 mg) or a surgically excised sample (1 g) of the viable tumor portion confirmed by the pathologist. Blood (10 mL for plasma) will be drawn when a medically required blood draw is present. Midstream urine (10 mL) and fecal (2–3 cc) samples will be collected from patients using the information leaflet provided. Samples will be collected 3–4 times, and an overview of the planned sample collection is shown in Tables 2, 3, and 4. All samples will be anonymously labeled with a pseudo-patient study identifier.

**Table 2:** Study schedule of visits, and sampling of upfront cytoreductive nephrectomy group (Group A)

Time	0	*X*	*X* + 4 months	*X* + 7 months
Visit	Baseline visit	During surgery	After ICI induction phase (4 cycles)	After ICI maintenance phase (6 cycles)
Blood sample	O	O	O	O
Urine sample	O	O	O	O
Stool sample	O	O	O	O
Tumor tissue sample	O	O

**Table 3:** Study schedule of visits, and sampling of deferred cytoreductive nephrectomy group (Group B)

Time	0	*X*	*X* + 3 months	*X* + 4 months	*X* + 7 months
Visit	Baseline visit	Before ICI therapy	After ICI induction phase (4 cycles)	During surgery	After ICI maintenance phase (6 cycles)
Blood sample	O		O	O	O
Urine sample	O		O	O	O
Stool sample	O		O	O	O
Tumor tissue sample	O			O

**Table 4:** Study schedule of visits, and sampling of immune checkpoint inhibitor therapy group (Group C)

Time	0	*X*	*X* + 3 months	*X* + 6 months
Visit	Baseline visit	Before ICI therapy	After ICI induction phase (4 cycles)	After ICI maintenance phase (6 cycles)
Blood sample	O		O	O
Urine sample	O		O	O
Stool sample	O		O	O
Tumor tissue sample	O			

### Public and patient involvement statement

Neither public nor patients were involved in the protocol development and study design.

### Sample size justification

Two prospective RCTs, PROBE (NCT04510597) and NORDICSUN (NCT03977571), are currently ongoing, but do not include an upfront CN arm. We determined the sample size based on the most recent real-world data that analyzed the intermediate/high risk group of mRCC patients stratified into upfront CN, deferred CN, and systemic treatment group [17]. To determine the required sample size for the study, we utilized two calculators for two-group survival analysis [18]. For Calculator 1, with a two-tailed alpha level (*α*) set at 0.05 and a beta level (*β*) of 0.2, along with a proportion of subjects in Group 1 (upfront and deferred CN) of 0.66 and a proportion in Group 0 (ICIs only) of 0.34, the relative hazard was set at 0.37. The calculated number of events needed to achieve 80% power was 35. For Calculator 2, with the total number of events set at 35, and assuming a baseline event rate of 0.53 and a median survival time in Group 0 (ICIs only) of 1.3 years, along with a censoring rate of 0.1 and a planned average length of follow-up of 5 years, the sample sizes required for each group were determined. Therefore, a total of 57 subjects were needed for the study.

### Statistical analysis

Descriptive statistics will be used to assess clinicopathological characteristics. Mean values and standard deviation will be provided for continuous variables, whereas frequency and percentage distributions will be provided for categorical variables. Groups will be compared using the chi-square test for categorical variables and the Student’s *t*-test for continuous variables. The Kaplan–Meier and log-rank test method and log-rank test will be used to estimate and compare OS and PFS. Univariate and multivariate survival analyses will be conducted using the Cox proportional hazards model, which will provide hazard ratios (HRs) with 95% confidence intervals (CI). For subjects without endpoints or who violate the study protocol will be excluded. Statistical analysis will be performed using SPSS software (version 25.0; IBM, Armonk, NY) and R software [V.3.6.2 (http://www.R-project.org)]. The significance level will be set at *P* < 0.05, and all statistical tests will be two-sided.

Microbiome analyses will be conducted using 16S rRNA sequencing to determine the differences in alpha diversity among the three groups by calculating the Shannon, Chao1, and Simpson indices. Beta diversity will be generated using the weighted UniFrac distance and visualized using a principal component analysis plot. Between-group differences were analyzed using multivariate analysis of variance with permutation. Furthermore, subgroup analysis according to the number and location of metastasis will be performed.

### Oversight and monitoring

We have composed a study coordinating center and data management and monitoring team. The study coordinating center is composed of a project general manager (JSP) and project manager at each institution (JK, and JJ) which decides on the study protocol and performance after internal discussion and has responsibility for each study process. The data management and monitoring team consists of WSJ and WKH, which is independent of competing interests, will be composed to monitor data safety and access to the final dataset.

All patient data collected and processed will be managed by the investigators with adequate precautions to ensure the confidentiality of the data. All samples will be saved and stored in a pseudonymized form.

### Interim analyses

The data management and monitoring team monitors safety outcomes and provides recommendations regarding the continuation or premature termination of the trial. No interim analyses concerning efficacy are performed. We planned two interim statistical analyses on safety during the course of this study, after approximately 25% and 50% of the inclusion. The only stopping condition is based on safety. The decision to stop or continue is made by the trial team based on the advice of the data management and monitoring team. The risk of life-threatening immune-related adverse complications will be evaluated.

### Data management and checks

The data management and monitoring team will be responsible for overseeing the receiving, entering, cleaning, querying, analyzing, and storing of all data that accrues from the study by designated persons. Each month, this team will randomly check the files in all three centers. Furthermore, daily monitoring of the data will be done by the members of the study coordinating center to minimize entry errors. All data will be stored on access-controlled computers and servers in line with data security policies. Databases will be backed up monthly.

### Adherence with study protocol

Non-adherence with the trial protocol will be reported and will be dropped out from the study.

### Missing data

Missing data will be minimized by performing a thorough data cleaning process until data are either received, confirmed as unavailable or the trial has reached the analysis stage. If missing for less than 5% of participants, these missing values will be imputed using mean or median values (depending on the distribution of non-missing data) for the whole cohort. If missing for more than 5% of participants, then multiple imputation will be considered.

## Ethics and dissemination

### Ethics

The study has been approved by the Institutional Review Board (IRB) of the YUHS (approval nos.4–2022-1453, 2023–0318-001, 2022–0618-001) and will be performed in accordance with the principles enunciated in the current version of the Declaration of Helsinki. Each substantial protocol amendment will be notified for approval to the IRB of the YUHS prior to implementation.

### Dissemination plan

Upon completion of the study, we intend to present the results as oral communications and abstracts at national and international urological meetings. The results will be published in a peer-reviewed journal. Publication policy of this trial has been negotiated and specified in contractual obligations and agreements between involved investigators.

## Discussion

The optimal use of CN in mRCC settings remains unclear, especially in ICI therapy settings [19], where the most recent evidence has been generated in the TKI era [19]. Although the potential of CN in the primitive IO era has drawn attention, no studies have thoroughly evaluated the role of CN in ICI therapy in patients with mRCC, where recent guidelines have moved away from TKI monotherapy in favor of ICI-based combinations. This is the first study to comprehensively incorporate both upfront and deferred CN and compare both with ICI therapy, redefining the role and timing of surgeries in mRCC. Because oncologic surgery can effectively control cancer by removing tumor cells [20], it should be considered as a treatment option for mRCC in selected patients, weighing the benefits and risks tailored to each patient. Moreover, CN may reduce the cost of ICI therapy, allowing for therapeutic holidays [19]. As patient selection is critical for expanding the role of CN in the contemporary IO era [21], the objective of this study is to develop biomarkers for patient selection for CN by analyzing gene mutation profiles and microbiomes. Due to the small number of study population, there could be some difficulties in drawing conclusions for discovery of biomarkers. Therefore, we will first achieve our primary endpoint, OS, and will expand the study for other analysis. Furthermore, the decision to perform CN should be discussed by a multidisciplinary team and clearly explained to the patients during shared decision-making sessions.

This study was registered at ClinicalTrials.gov on March 3, 2023. Patient accrual began in December 2022. Follow-up will be performed for at least 5 years or until death.

## Trial status

Recruitment of participants started in July 2023 and will be completed in December 2027. The manuscript reports protocol version 1.0 (January 2, 2023).

### Supplementary Information

Additional file 1: SPIRIT checklist.

Additional file 2: Patient consent form.

## Data Availability

The datasets generated and/or analyzed during the current study are available from the corresponding author on reasonable request.

## Abbreviations

CN: Cytoreductive nephrectomy
RCC: Renal cell carcinoma
mRCC: Metastatic renal cell carcinoma
IO: Immuno-oncology
CPI: Checkpoint inhibitor
IMDC: International mRCC Database Consortium
ST: Systemic therapy
ICIs: Immune checkpoint inhibitors
TKI: Tyrosine kinase inhibitor
RCTs: Randomized clinical trials
OS: Overall survival
PFS: Progression-free survival
ORR: Objective response rate
TIME: Tumor immune microenvironment
YUHS: Yonsei University Health System
SPIRIT: Standard Protocol Items: Recommendations for Interventional Trials
RECIST: Response evaluation criteria for solid tumors
INR: International normalized ratio
ULN: Upper limit of normal
NYHA: New York Heart Association
HIV: Human immunodeficiency virus
AIDS: Acquired immunodeficiency syndrome
CTCAE: Common Terminology Criteria for Adverse Events
HRs: Hazard ratios
CI: Confidence intervals
IRB: Institutional Review Board
