# Supplementary material for: The role of cytoreductive nephrectomy in metastatic renal cell carcinoma in immune-oncology era (SEVURO-CN): study protocol for a multi-center, prospective, randomized trial
Source: Trials. 2024 Jul 3;25:447. doi: 10.1186/s13063-024-08234-2 (PMC11223430; doi:10.1186/s13063-024-08234-2)
Supplement: Supplementary file 2 — Additional file 2: Patient consent form. [file 13063_2024_8234_MOESM2_ESM.doc]

**Patient Consent Form**

| **Study Title:** | The role of cytoreductive nephrectomy in metastatic renal cell carcinoma in immune-oncology era |
| --- | --- |
| **Project Investigator:** | Department of Urology, Severance Hospital, Yonsei University Health System, Professor Won Sik Ham |

This explanation has been prepared to help you understand this study, and contains detailed information about this study. Please read this explanation, fully understand, think about it, and then decide whether or not to participate.

Research is conducted with the purpose of finding out what has not yet been clearly identified. Therefore, before deciding whether to participate in this study, you should be informed of the purpose and procedures of this study, who will participate in this study, and what benefits and risks or inconveniences you can expect from participating in this study. It is important to hear and understand the research fully.

If you wish, you may discuss this with your family or other people. If you decide to participate in this study, please indicate your consent on the consent form.

You may participate in the study if you voluntarily agree and complete a consent form, or you may decide not to participate in this study. If you decide not to participate, it will not affect the treatment you will receive or result in any other disadvantages.

**1. Background and objectives of this study**

Renal cell carcinoma accounts for approximately 3% of all cancers worldwide, and in Korea, based on 2018 statistics, it accounts for 2.2% of all cancers in Korea and has the second highest prevalence among urinary tract cancers. Moreover, about 1/3 of renal cell carcinomas are found to have metastasized at the time of diagnosis, and about 1/3 of them have a poor prognosis, such as recurrence or progression after surgery. When analyzing the incidence rate of renal cell cancer by stage over the past 5 years in Korea, localized renal cell carcinoma accounted for 72.8%, metastatic renal cell carcinoma accounted for 27.2%, of which locally advanced cancer accounted for 10.8% and distant renal cell cancer accounted for 27.2%. Metastases were reported in 11.1%. In the case of metastatic renal cell carcinoma, international guidelines mainly recommend systemic therapy based on targeted therapy, immunotherapy, or a combination of the two drugs. In case of surgical treatment in addition to drug treatment, cytoreductive nephrectomy and metastasis resection are recommended for some selected patients.

Cytoreductive nephrectomy is performed on the principle of increasing response to systemic therapy by removing the primary lesion, reducing the number of systemic lesions during systemic treatment and preventing further metastasis to other organs, and was originally performed with primitive immuno-oncology drug, interferon-alpha, developed a long time ago. Although not as effective as current immuno-oncology drugs, this systemic treatment was effective when combined with cytoreductive nephrectomy. However, with the rapid development of targeted therapies and the emergence of new next-generation immunotherapy agents, questions have arisen as to whether cytoreductive nephrectomy will continue to be necessary for metastatic renal cell carcinoma. There have been two large prospective studies evaluating the effectiveness of cytoreductive nephrectomy when using targeted therapy, and the results of both studies have downsized the role of cytoreductive nephrectomy. However, the follow-up results that have recently been published suggest that cytoreductive nephrectomy may be effective in some patients, and it is controversial about the effectiveness of cytoreductive nephrectomy, the timing of surgery, and selection of patients for surgery. In addition, the above studies are all studies that used targeted therapy, and there are no prospective studies on cytoreductive nephrectomy in association with immunotherapy agents. Recently published retrospective studies have shown that cytoreductive nephrectomy is effective when combined with immunotherapy agents. Cytoreductive nephrectomy reduces the number of systemic lesions during systemic treatment by removing the primary lesion, prevents additional metastasis to other organs, and increases response to systemic therapy. As a result of our research team's animal experiments, it has been proven that cytoreductive nephrectomy prevents immune cells from being exhausted by removing the primary cancer which reduces various substances that interfere with immune function thereby increasing the efficacy of next-generation immunotherapy agents.

However, there are no reports on prospective clinical trials regarding cytoreductive nephrectomy in association with immunotherapy. There are currently two prospective studies underway, but both of these studies only included deferred cytoreductive nephrectomy not upfront cytoreductive nephrectomy. This study aims to evaluate the role of both upfront and deferred cytoreductive nephrectomy in association with immunotherapy in Korean patients with metastatic renal cell carcinoma. We aim to identify for the role of cytoreductive nephrectomy in the era of immuno-oncology drugs, by comparing upfront and deferred cytoreductive nephrectomy, and immune-oncology drugs.

In addition, we are planning to perform genetic analysis and microbial analysis in tumor tissue, blood, urine, and stool of metastatic renal cell carcinoma patients in order to evaluate treatment response, recurrence, and resistance to the treatment.

**2. Number, period and location of subjects participating in this study**

This study will involve a total of 57 patients at Severance Hospital and will be conducted for over 10 years.

If you agree to participate in the study, only the hospitalization and outpatient visits are required for surgery and drug treatment, and no additional hospitalization or outpatient visits are necessary.

This study targets patients who were diagnosed with metastatic renal cell carcinoma and scheduled for treatment at Severance Hospital who voluntarily completed the consent form and provided human-derived material by agreeing to this study. Human-derived material refers to human body components such as cells, tissues, body fluids, blood, urine, feces, etc. collected during treatment (cell examination, tissue examination, surgery, drug treatment, and etc.) or materials separated from them (serum, plasma, chromosomes, DNA, RNA, proteins, and etc.) in accordance with the Acts on Bioethics and Safety.

Human-derived materials collected during your treatment are primarily used for diagnosis, monitoring diseases, and establishment of treatment plan. Please donate your human-derived materials and related information for our researches to understand the causes of the diseases, and development of new treatment modality, preventive measures and prognostic factors for metastatic renal cell carcinoma. It will be of great use. This study is scheduled to enroll 57 patients over 10 years after IRB approval.

**3. Procedures and methods**

To evaluate the role of cytoreductive nephrectomy in the era of immunotherapy, which is the primary goal of this study, patients will be divided into three treatment groups, as follows.

1. Upfront cytoreductive nephrectomy

2. Deferred cytoreductive nephrectomy

3. Immune checkpoint inhibitors (Immuno-oncology drugs) only

The upfront cytoreductive nephrectomy group will undergo cytoreductive nephrectomy (±metastasectomy) before immunotherapy, and the deferred cytoreductive nephrectomy group will undergo cytoreductive nephrectomy (± metastasectomy) after immunotherapy. The immune checkpoint inhibitors (Immuno-oncology drugs) only group will receive only immunotherapy.

Here are the steps you will follow after you agree to participate in this study.

After determining whether the patient is possible participate in this study, a decision will be made as to whether surgery is possible. If surgery is possible, patients will receive postoperative drug treatment or drug treatment alone according to 1:1 random allocation. If surgery is not possible, drug treatment will be given first.

Surgical treatment involves cytoreductive nephrectomy and metastasectomy. Cytoreductive nephrectomy is a process of removing the primary cancer originated from kidney and metastatic lesions as much as possible.

In the case of drug monotherapy, combination immunotherapy (nivolumab + ipilimumab) is given according to existing treatment guidelines, and after 4 cycles of treatment at 3-week intervals (total 12 weeks = 3 weeks/cycle x 4 cycles; induction phase), drug treatment is completed. Response to the treatment will be evaluated. In the group that received surgical treatment, drug treatment will be administered within 6 weeks after surgery (total 12 weeks = 3 weeks/cycle x 4 cycles). Patients who have received drug treatment alone will be evaluated to see if surgical treatment is possible. If surgical treatment is possible, they will receive surgery or drug treatment by 1:1 random allocation. If surgery is not possible, drug treatment will be maintained. If you undergo surgical treatment after drug treatment, the surgery will be performed within 6 weeks. Patients who maintain drug treatment will receive 6 cycles of treatment (maintenance phase, nivolumab only) at 2-week intervals, and response to drug treatment will be evaluated every 3 months. According to existing drug treatment guidelines, treatment is discontinued when the introduction and maintenance periods exceed 2 years.

In the case of human-derived materials obtained during the treatment process, approximately 20 mg of tumor tissue is collected from the biopsy performed when diagnosing metastatic renal cell carcinoma, and in the case of surgical patients, approximately 1 g of tissue that does not interfere with the diagnostic purpose is collected. During the course of treatment, 10 cc each of blood and urine collected every 3-4 months will be additionally collected during the collection of tests required during follow-up, and there will be no in-office procedure for a separate collection process due to this study. 2-3 cc of stool is collected and requires separate collection, but the collection process itself is not difficult and a kit for collection is provided. A total of 3-4 samples (blood, urine, stool) will be collected during follow-up.

Participation in this study will shed light on the role of cytoreductive nephrectomy during immunotherapy treatment, identify groups that require surgical treatment, and help determine the timing of surgical treatment in those groups. In addition, through genetic analysis and microbial genome analysis of tissues, blood, urine, and stool of metastatic renal cell carcinoma patients, the role of cytoreductive nephrectomy in immunotherapy treatment and prediction of response, recurrence, and resistance to determination of resistance after drug and surgical treatment will be evaluated.

Please read and consider each of the following items to decide whether to provide your human-derived material.

1. Your personal information related to this study at the Severance Hospital will be kept anonymous and will not be transferred, and medical information required for research, such as future clinical information, will be handled by the Institutional Review Board (IRB). It may be used for research purposes with approval.

2. Research results using information related to human-derived material may be published under the name of the researcher at a conference or academic journal, and your identity will not be revealed during this process.

3. Future research using your human-derived material will be conducted after review and approval by the IRB. The types of research in which human-derived material can be used are varied and may include genetic research. However, the specific nature and purpose of the study is unknown at this time.

4. If, as a result of your research using human-derived materials, there is development of a product such as a new drug or diagnostic tool or application for a patent, you cannot claim rights regarding this.

5. You may refuse the contents of this consent form, and even after giving consent, your consent will be continuously confirmed verbally during outpatient visits. If you wish to withdraw, you may leave a written justification and cancel in writing at any time.

6. Your human-derived material will be preserved for 10 years at Severance Hospital, and any human-derived material that has expired will be disposed of in accordance with the methods and procedures set forth in the Bioethics and Safety Acts.

7. Collected human-derived materials cannot be used for purposes other than research and are not provided to third parties.

If you decide to participate in this study, you will sign a informed consent form.

**4. Benefits that can be expected from participating in this study**

When you participate in the study, samples are collected in addition to routine examination. Therefore, there is no particular risk to the patient. Based on the results of this study, the role of cytoreductive nephrectomy in immunotherapy for patients with metastatic renal cell carcinoma will be confirmed. It can help identify groups that require surgical treatment and determine the timing of surgical treatment in those groups. In addition, it can provide useful information to patients and clinicians by making it possible to determine the diagnosis, recurrence, and resistance of metastatic renal cell carcinoma through human-derived materials. However, there may be no direct benefit to the subject.

**5. Expected risks and inconveniences from participating in this study**

∙ In two large-scale prospective studies evaluating the effectiveness of cytoreductive nephrectomy when using targeted therapy, when cytoreductive nephrectomy was performed before using targeted therapy, 10-20% of subjects did not receive chemotherapy due to disease progression or deterioration of physical condition. There were cases in which surgery was not performed, but in those studies, relatively high-risk groups were included in the studies, so the risk of surgery may have been evaluated as high, and recent studies have shown that upfront cytoreductive nephrectomy may be effective in certain groups. It was reported that the oncological prognosis was good in the group that underwent upfront cytoreductive nephrectomy. However, due to situations such as unexpected deterioration in physical condition due to surgery, the administration of immunotherapy may not be possible.

∙ The cost of storage and management of collected human-derived materials and tests performed for future research is borne by the researcher, and there are no additional tests or costs to be paid by the subject. There are very few risks, side effects, or injuries associated with your participation in the study.

**6. Costs of participating in this study**

There are no separate costs for you by participating in this study.

**7. Compensation for participation in this study**

There will be no separate compensation fee for your participation in this study.

**8. If damage related to research occurs, compensation or treatment to be given to the subject.**

The procedures of this study do not deviate from the normal course of care you would experience even if you were not participating in the study. Therefore, you will be subject to safety protection measures taken in the normal course of treatment, and you will be provided with sufficient information about the purpose and methods of the study before starting the study. If, while participating in a clinical study, expected or unexpected adverse reactions occur due to procedures or interventions outside of the existing medical treatment process, we will provide the best possible treatment to ensure that you receive appropriate treatment.

**9. Matters that the subject must comply with**

There are no special requirements for you to comply with by participating in this study.

**10. Dropping out of study participation**

You may stop participating in the study at any time, even after giving consent.

**11. Collection and provision of information**

By signing this consent form, you consent to the researchers collecting and using your personal (sensitive) information and, if you consent, this information may be provided to third parties.

1) Purpose of collection and use of personal information

Using the patient's personal information, we intend to evaluate the role of cytoreductive nephrectomy during immunotherapy treatment in patients with metastatic renal cell carcinoma, and use the analysis of the patient's human-derived materials to evaluate treatment response and recurrence and predict resistance after treatment.

2) Items of personal (sensitive) information wish to collect

Your name, gender, age, height, weight, and hospital registration number

3) Period of retention and use of personal information

Your personal (sensitive) information will be stored for 10 years and will be destroyed directly by the research director or research staff after the storage period.

4) The fact that you have the right to refuse consent and, if there are any disadvantages due to refusal of consent, the details of those disadvantages

You are free to decide whether or not to accept the collection, use, and provision of the above personal (sensitive) information. Even if you do not accept the collection, use, or provision of personal (sensitive) information, there will be no disadvantage to your treatment or prescription.

5) Whether the personal information collected in the study is provided to others* and, if so, whether it includes personally identifiable information

The above personal (sensitive) information collected in this study will not be used for any secondary purposes other than the purpose of this study.

Your personal (sensitive) information will not be provided to third parties.

Whether or not you accept the provision of personal (sensitive) information can be indicated in the ‘Consent Form’ on the last page.

**12. Confidentiality of personal information and records**

Your records collected while you participate in this study will be kept confidential, and any records that identify you will remain confidential even if the results of the study are written, published, or announced in a report. no see.

Those who monitor/check this research, the Research Review Committee, and related ministries (e.g., Ministry of Health and Welfare), etc., will verify the reliability of the research procedures and data within the scope set by relevant regulations without violating your confidentiality. You can view your medical records directly. By signing this consent form, you authorize yourself or your legal representative to view these materials.

**13. Voluntary Participation/Withdrawal**

You may withdraw your consent to participate in the clinical study at any time, in which case your participation in the clinical study will end and the researchers will not collect any additional information from you related to the study.

If you do not want the collected information and human-derived material to be destroyed and used, you must contact the researcher and convey your wishes.

Your decision will not affect the treatment you will receive in the future.

If you decide not to participate or to withdraw from the study, you will not be penalized in any way and you will not lose any benefits to which you were originally entitled.

If new information is collected that may affect your decision to continue participating in the study, the researcher will inform you or your representative of this information in a timely manner.

**14. Contact information**

If you have any questions about this study or if an injury related to the study occurred, please contact the researcher below.

**Researcher's name: Won Sik Ham**

**Researcher Address: 50-1 Yonsei-ro, Seodaemun-gu, Seoul, 120-752**

**☎ 02-2228-2310**

If you have any questions about your rights, you may speak to the researcher or call the number below.

**Severance Hospital Research Review Committee ☎ 02-2228-0430~4**

**Severance Hospital Clinical Research Protection Center ☎ 02-2228-0450~4**

**Patient consent form**

| **Research title :** | | The role of cytoreductive nephrectomy in metastatic renal cell carcinoma in immune-oncology era | | | | | |  |
| --- | --- | --- | --- | --- | --- | --- | --- | --- |
| **※ If you have read and agree to the items below, please check the box on the left.** | | | | | | | |  |
| □  □ | I have read this explanation and have fully read and understood the purpose, method, expected effects, possible risks, presence and content of other treatment methods, health information management, and etc. of this clinical study.  For the purpose of this research, I have understood the explanations regarding the collection, use, and provision of personal (sensitive) information.  • Do you consent to providing your personal information for purposes other than those described in the statement?  ꠓ □ **Yes** □**ꠓ No**  (If “Yes,” please indicate whether you agree or disagree with the items below.)  • Do you agree to include personally identifiable information when providing your personal (sensitive) information?  □ **Yes** □**ꠓ No** | | | | | | |  |
| □ | I asked all my questions and received sufficient answers. | | | | | | |  |
| □ | It has been confirmed that even if you consent to this study, you may withdraw your consent at any time and receive other appropriate treatment after withdrawal. | | | | | | |  |
| □ | I will receive a copy of the explanation and completed consent form. | | | | | | |  |
| □ | After taking sufficient time and consideration, I freely agree to participate in this study. | | | | | | |  |
| Subject name | | |  | Signature |  | Signature date |  | |
|  | | | | | | | | |
|  | | | | | | | | |
| * Representative Name  *(If necessary)* | | |  | Signature |  | Signature date |  | |
| (Relationship with the subject: ) | | | | | | | | |
|  | | | | | | | | |
| Watcher name  *(If necessary)* | | |  | Signature |  | Signature date |  | |
|  | | | | | | | | |
| Name of researcher described | | |  | Signature |  | Signature date |  | |
